# *Helicobacter pylori* infection exacerbates metabolic dysfunction-associated steatotic liver disease through lipid metabolic pathways: a transcriptomic study

**DOI:** 10.1186/s12967-024-05506-y

**Published:** 2024-07-29

**Authors:** Xingcen Chen, Ruyi Peng, Dongzi Peng, Deliang Liu, Rong Li

**Affiliations:** 1 Department of Gastroenterology, The Second Xiangya Hospital of Central South University, No. 139 Middle Renmin Road, Changsha, 410011 Hunan Province China; 2 Research Center of Digestive Diseases, Central South University, No. 139 Middle Renmin Road, Changsha, 410011 Hunan Province China; 3 Clinical Research Center for Digestive Diseases in Hunan Province, Changsha, 410011 Hunan Province China

**Keywords:** *Helicobacter pylori*, Metabolic dysfunction-associated steatotic liver disease, Transcriptome sequencing, FABP5, PPAR signaling pathway

## Abstract

**Background:**

The relationship between *Helicobacter pylori* (*H. pylori*) infection and metabolic dysfunction-associated steatotic liver disease (MASLD) has attracted increased clinical attention. However, most of those current studies involve cross-sectional studies and meta-analyses, and experimental mechanistic exploration still needs to be improved. This study aimed to investigate the mechanisms by which *H. pylori* impacts MASLD.

**Methods:**

We established two *H. pylori*-infected (Cag A positive and Cag A negative) mouse models with 16 weeks of chow diet (CD) or high-fat diet (HFD) feeding. Body weight, liver triglyceride, blood glucose, serum biochemical parameters, inflammatory factors, and insulin resistance were measured, and histological analysis of liver tissues was performed. Mouse livers were subjected to transcriptome RNA sequencing analysis.

**Results:**

Although *H. pylori* infection could not significantly affect serum inflammatory factor levels and serum biochemical parameters in mice, serum insulin and homeostatic model assessment for insulin resistance levels increased in CD mode. In contrast, *H. pylori* Cag A + infection significantly aggravated hepatic pathological steatosis induced by HFD and elevated serum inflammatory factors and lipid metabolism parameters. Hepatic transcriptomic analysis in the CD groups revealed 767 differentially expressed genes (DEGs) in the *H. pylori* Cag A + infected group and 1473 DEGs in the *H. pylori* Cag A- infected group, and the “nonalcoholic fatty liver disease” pathway was significantly enriched in KEGG analysis. There were 578 DEGs in *H. pylori* Cag A + infection combined with the HFD feeding group and 820 DEGs in the *H. pylori* Cag A- infected group. DEGs in the HFD groups were significantly enriched in “fatty acid degradation” and “PPAR pathway.” Exploring the effect of different Cag A statuses on mouse liver revealed that fatty acid binding protein 5 was differentially expressed in Cag A- *H. pylori*. DEG enrichment pathways were concentrated in the “PPAR pathway” and “fatty acid degradation.”

**Conclusions:**

Clinicians are expected to comprehend the impact of *H. pylori* on MASLD and better understand and manage MASLD. *H. pylori* infection may exacerbate the development of MASLD by regulating hepatic lipid metabolism, and the *H. pylori* virulence factor Cag A plays a vital role in this regulation.

**Supplementary Information:**

The online version contains supplementary material available at 10.1186/s12967-024-05506-y.

## Introduction

*Helicobacter pylori* (*H. pylori*) infects approximately 4.4 billion people worldwide, with a prevalence of 43.1% (40.3–45.9) [1, 2]. A family-based epidemiological survey revealed that the prevalence of *H. pylori* infection in China is approximately 40.66%, 43.45% in adults, and 20.55% in children and adolescents [3]. Multitudinous studies have confirmed that *H. pylori* infection is an essential factor in the progression from gastritis to gastric cancer [4, 5]. Cytotoxin-associated gene A (Cag A) is considered the most vital virulence factor of *H. pylori*, and several studies have shown that Cag A is directly associated with DNA damage in gastric epithelial cells and gastric mucosal carcinogenesis [6, 7]. In addition to gastritis, gastric ulcers, and gastric cancer, many extra-gastric diseases, such as atherosclerosis, Parkinson’s disease, and metabolic dysfunction-associated steatotic liver disease (MASLD), are also closely associated with *H. pylori* infection [8].

MASLD is a clinicopathologic syndrome characterized by excessive fat deposition in hepatocytes while meeting one of overweight or obesity, type 2 diabetes mellitus, or metabolic dysfunction [9]. The disease spectrum includes simple hepatocellular steatosis, metabolic dysfunction-associated steatohepatitis (MASH), MASH-related liver fibrosis, and hepatocellular carcinoma (HCC). The pathogenesis of MASLD remains unknown, and the *multiple-hit* theory reviewed by Buzzetti et al. is widely acknowledged in academia [10]. MASLD has become the most common chronic liver disease worldwide, with a global prevalence of approximately 32.4% (29.9–34.9) [11, 12]. Although MASLD is an urgent public health problem, countries still need to fully prepare to address it [13]. No effective agents have been approved for MASLD treatment, and the primary clinical management regimen for MASLD is to identify patients with a high risk of disease progression and lose weight through dietary modification and physical exercise [11]. It is pressing to recognize and manage MASLD correctly.

Since the first report of *H. pylori* DNA detected in the livers of MASLD patients [14], several clinical studies have focused on the relationship between *H. pylori* infection and MASLD. Many researchers discuss the relationship between the two and believe that *H. pylori* infection may be used as a combustion aid in the *multiple-hit* theory of MASLD, exacerbating the progression of MASLD through the aspects of inflammatory factors, adipokines, the intestinal barrier, and the intestinal flora [15, 16]. Yu et al. substantiated that eradication of *H. pylori* in *H. pylori*-positive MASLD patients ameliorated fasting blood glucose (FBG), serum triglyceride (TG), insulin resistance (IR), and body mass index (BMI) [17]. The study by Abdel-Razik et al. reached similar conclusions [18]. However, other studies found no association between *H. pylori* infection and MASLD. A Mendelian randomization study by Liu et al. revealed no causal link between *H. pylori* infection and MASLD and no significant association between *H. pylori* infection and TG, high-density lipoprotein cholesterol (HDL-C), low-density lipoprotein cholesterol (LDL-C), or FBG levels [19]. Interestingly, a cross-sectional study by Kang et al. indicated that Cag A status may be critical to influencing the relationship between the two, and there was no association between the Cag A positive (Cag A+) *H. pylori* group and MASLD (OR: 1.05; 95% CI: 0.81–1.37), and in multivariate analysis, the Cag A negative (Cag A-) *H. pylori* group was significantly associated with MASLD (OR: 1.30; 95% CI: 1.01–1.67) [20]. Therefore, this study aimed to explore the effect of *H. pylori* infection with different Cag A status on the liver under different dietary patterns and the relationship between *H. pylori* infection and MASLD.

## Methods

### *H. pylori* culture

The rodent-adapted *H. pylori* Sydney strain (SS1) (Cag A+) was donated by Professor Yong Xie (Department of Gastroenterology, First Affiliated Hospital of Nanchang University, Jiangxi, China). *H. pylori* Cag A- was isolated from gastric ulcer patients’ specimens via gastroscopy. The *H. pylori* strains grown on Columbia blood agar plates supplemented with antibiotics (10 mg/L vancomycin, 5 mg/L cefsulodin, 5 mg/L amphotericin B, and 5 mg/L trimethoprim) and 10% sheep blood (Bianzhen Biotech, Nanjing, China) at 37 °C under microaerophilic conditions (5% O_2_, 10% CO_2_, and 85% N_2_) for 3–4 days. Then, the *H. pylori* strain, which was in the early log phase with good motility and activity for subculture or intervention, was harvested and resuspended in phosphate buffer saline (PBS). The *H. pylori* concentration was estimated by measuring the OD_600 nm_, where OD_600 nm_ corresponds to approximately 2 × 10^8^ colony-forming units (CFU)/ml.

### Animals and treatment

All animal studies were performed according to the National Institutes of Health recommendations for the Care and Use of Laboratory Animals and approved by the Central South University Animal Ethics Committee. Male C57BL/6J mice (specific pathogen-free grade) aged 6–8 weeks were purchased from Hunan SJA Laboratory Animal Co., Ltd and housed in animal quarters at 20–22 ° C with a 12-h light cycle and fed ad libitum. After one week of adaptive feeding, 48 mice were randomly divided into six groups (CD + PBS, CD + *H. pylori* Cag A+, CD + *H. pylori* Cag A-, HFD + PBS, HFD + *H. pylori* Cag A+, and HFD + *H. pylori* Cag A-) of 8 mice each. Four groups were intragastrically infused seven times with 1 × 10^9^ CFU of *H. pylori* Cag A + or *H. pylori* Cag A- at 1-day intervals and fed either a regular chow diet (CD) or a high-fat diet (HFD) (Research Diets, D09100310). Caloric composition of CD versus HFD is shown in Table 1. At the same time, the other two groups received PBS by gavage and were fed the corresponding diet for 16 weeks. Mice were fasted overnight prior to sacrifice. Six animals per group were used for testing and statistical analysis.

**Table 1:** Caloric composition table of mouse diet

Dietary component	Protein	Carbohydrate	Fat	Calories
Chow diet	22.9%	66.0%	11.1%	3.37 Kcal/gm
High fat diet	20.0%	40.0%	40.0%	4.49 Kcal/gm

### Biochemical analysis

Serum alanine aminotransferase (ALT), aspartate aminotransferase (AST), total cholesterol (TC), HDL-C, and LDL-C were assayed by an automatic biochemical analyzer (Rayto Life and Analytical Sciences, Chemray 240) with corresponding commercial kits. Enzyme-linked immunosorbent assay (ELISA) kits (Jiangsu Meimian Industrial, MM-0040M1, MM-0163M1, MM-0132M1, and MM-0579M1) were used to detect Interleukin 1β (IL-1β), Interleukin 6 (IL-6), Tumor necrosis factor α (TNF-α) and insulin levels in mouse serum. Hepatic TG was measured by a commercial kit (Nanjing Jiancheng Bioengineering Institute, A110-1-1) according to the manufacturer’s instructions.

### Histopathologic examination

Fresh mouse liver tissues were fixed in 4% paraformaldehyde and embedded in paraffin. Embedded tissues were cut at 4 μm thickness and then stained with a hematoxylin-eosin (H&E) kit (Powerful Biology, Wuhan, China) for histological assessment according to the rodent model MASLD scoring system proposed by Liang et al. [21]. Frozen samples were cut into 8-µm sections and stained with an Oil Red O staining kit (Powerful Biology, Wuhan, China) according to the manufacturer’s instructions. Masson staining was used to observe fibrosis in the liver of mice. After staining with iron hematoxylin, Ponceau, and aniline blue, collagen fibers were blue, and muscle fibers, cytoplasm, and cellulose were red. Immunohistochemical (IHC) staining was performed using the following methods: Paraffin slices, 4 μm thick, were grilled at 65 °C for 60 min, then dewaxed in xylene and rehydrated in a series of increasingly diluted ethanol. High-temperature antigen retrieval was achieved by microwave treatment in 0.1 M citrate solution (pH 6.0) for 10 min. The slices were treated with 3% H_2_O_2_ at room temperature for 20 min, followed by incubation with goat serum for 20 min, and subsequently with anti-FABP5 rabbit polyclonal antibody (Proteintech, Wuhan;1:100) overnight at 4 °C. The following day, the slices were brought to room temperature and incubated with the secondary anti-rabbit antibody for 20 min after washing with PBS. DAB coloration was applied, followed by mounting with hematoxylin and subsequent microscopic examination. All the samples were examined under a light microscope at 200× magnification.

### Intraperitoneal glucose tolerance test (IPGTT) and intraperitoneal insulin tolerance test (IPITT)

Sixteen hours of fasting and water deprivation preceded the IPGTT procedure, and the mice were injected intraperitoneally with glucose solution (2 g/kg body weight). Blood samples were collected by tail puncture at 0, 30, 60, 90, and 120 min to measure blood glucose levels using a glucometer. The area under the curve (AUC) was calculated for the IPGTT. IPITT was performed at three-day intervals. Mice were fasted for six hours and injected with insulin (0.75 IU/kg body weight) (Jiangsu Wanbang Biochemistry Medicine Co. Ltd., Xuzhou, China). Blood glucose levels were measured at 0, 15, 30, 60, and 90 min after insulin injection. The AUC of the IPITT was calculated.

### RNA‑sequencing

Total RNA preparation and subsequent RNA-seq library construction were performed using the APExBIO Technology LLC (Shanghai, China) service. Briefly, total RNA was isolated using a commercial kit (Tiangen Biotech, DP424), and RNA libraries were established using an RNA cleaning and concentration kit (APExBIO Technology LLC, K1159) after quality inspection and purity testing. The RNA quality was verified using an Agilent 2100 Bioanalyzer (Agilent Technologies, Santa Clara, CA). The qualified libraries were subjected to Illumina NovaSeq 6000 double-end sequencing according to the effective concentration and target data volume to obtain paired sequences with a read length of 150 bp. The filtered reads were mapped to the mouse genome reference sequence (GRCm39.dna.toplevel.fa Ensembl release103) using HISAT2. The gene expression levels were expressed as fragments per kilobase per million fragments (FPKM). Genes were considered differentially expressed when |fold change| >1.5 and P value < 0.05. Differential expression analysis was performed using DESeq2. KEGG and GO enrichment analyses of differentially expressed genes were performed using the R package ClusterProfiler (v4.2.2), and significant pathways were identified with a P value < 0.05.

### Quantitative real-time PCR (qRT-PCR)

Total RNA was extracted from the livers with AG RNAex Pro Reagent (Accurate Biotechnology, AG21101), an Evo M-MLV RT Mix Kit with gDNA Clean for qPCR Ver.2 (Accurate Biotechnology, AG11728) reverse-transcribed the extracted total RNA into cDNA. qRT-PCR was performed using the SYBR Green Premix Pro Taq HS qPCR Kit IV (Accurate Biotechnology, AG11746) and the targeting gene primers. All the gene primer sequences are shown in Table 2. PCR was performed in triplicate on the qRT-PCR detection system with the following cycling parameters: 95 °C (30 s), 40 cycles of 95 °C (5 s), 55 °C (30 s), and 72 °C (30 s). The qRT-PCR data were quantified by the 2^−ΔΔCt^ method.

**Table 2:** Sequences of primers used for qRT-PCR

Gene	Forward primer	Reverse primer
Gapdh	AGGTCGGTGTGAACGGATTTG	GGGGTCGTTGATGGCAACA
Fabp5	AGAGCACAGTGAAGACGAC	CATGACACACTCCACGATCA
Ppar-γ	TCGCTGATGCACTGCCTATG	GAGAGGTCCACAGAGCTGATT
Fgf21	CTGCTGGGGGTCTACCAAG	CTGCGCCTACCACTGTTCC
Srebf-1	GATGTGCGAACTGGACACAG	CATAGGGGGCGTCAAACAG
Il-1β	GAAATGCCACCTTTTGACAGTG	TGGATGCTCTCATCAGGACAG
Tnf-α	CCTGTAGCCCACGTCGTAG	GGGAGTAGACAAGGTACAACCC

### Statistical analysis

The data were expressed as the mean ± standard deviation (SD) and analyzed by unpaired student’s t-test and one-way analysis of variance (ANOVA) with GraphPad Prism 9.0 software. At least three independent biological replicates were performed for each group. *p* < 0.05 was considered statistically significant.

## Results

### *H. pylori* infection exacerbates hepatic lipid deposition and insulin resistance in mice

**Fig. 1:**
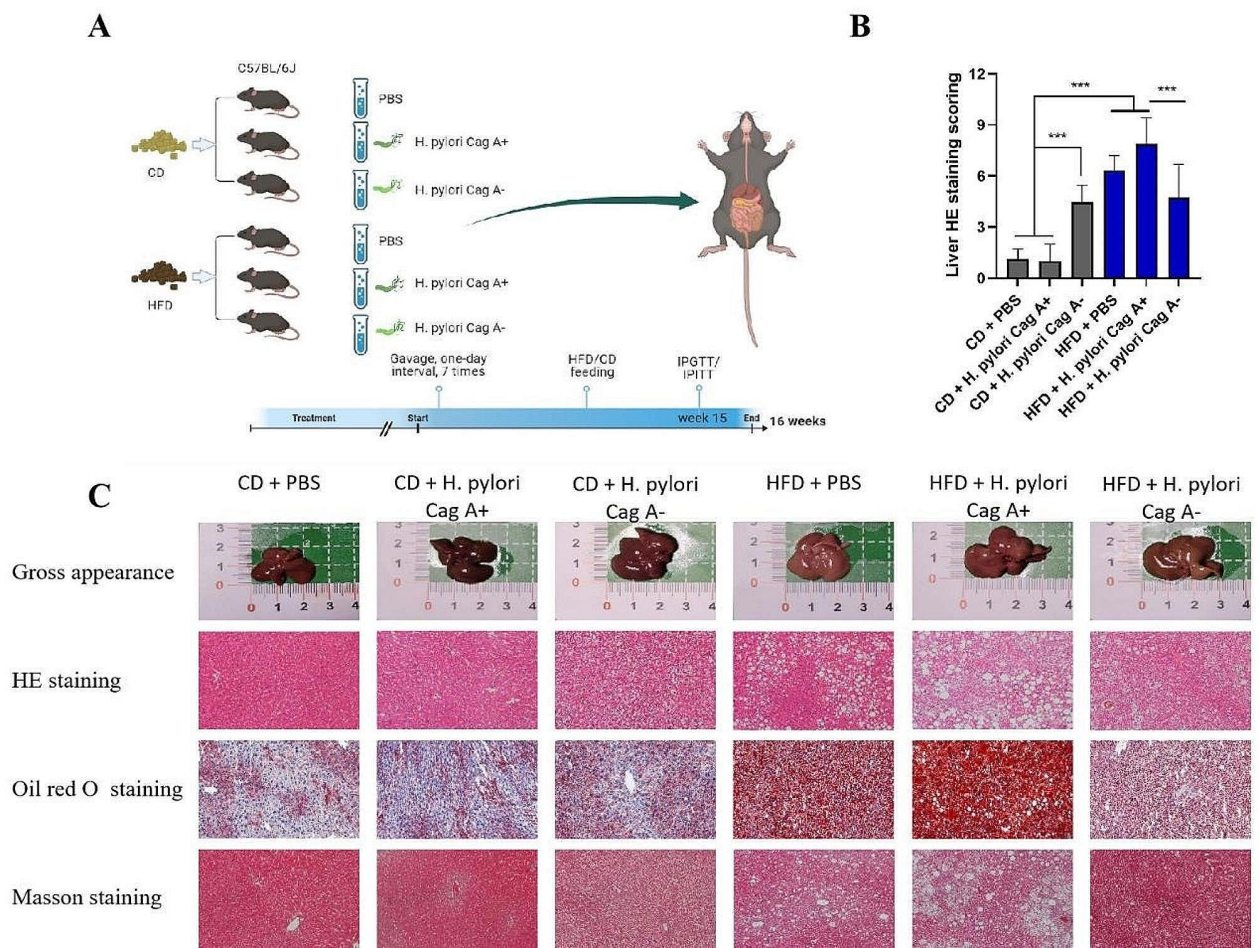
Effects of different *H. pylori* infection combined with CD/HFD feeding on liver pathology in mice. (**A**) Experimental protocol. (**B**) Semi-quantitative MASLD score by HE staining in CD/HFD groups. (**C**) Gross observation, HE staining, Masson staining and Oil Red O staining in CD/HFD groups, 200× magnification under a light microscope. Data are expressed as mean ± SD, *n* = 6, *P < 0.05, **P < 0.01, ***P < 0.001

The mice were sacrificed after 16 weeks of *H. pylori* infection and CD/HFD feeding (Fig. 1A). Giemsa staining, rapid urease test, and IHC staining with *H. pylori* antibody of mouse gastric mucosa tissues were conducted to confirm *H. pylori* infection (Supplementary Fig. 1A-B). In the CD groups, *H. pylori* infection did not significantly affect the mice’s body weight and hepatic TG (Supplementary Fig. 1^2^A, D). However, liver weight and liver weight ratio were significantly increased in mice infected with *H. pylori* Cag A- (Supplementary Fig. 2B, C). The mean body weight of the mice in the HFD groups was significantly greater than that of the CD groups. Interestingly, there was no significant difference in liver weight between HFD-fed mice and CD-fed mice; the liver weight ratio showed similar results. The hepatic TG content results more visually demonstrated the effect of HFD on hepatic lipid deposition, with the HFD + *H. pylori* Cag A + group having the most hepatic TG content (Supplementary Fig. 2D).

Different dietary patterns and different *H. pylori* strain infected mice exhibited different degrees of hepatocyte damage, such as hepatocyte vacuolation, lipid droplet deposition, and hepatocyte swelling, as shown by HE, Masson, and Oil Red O staining (Fig. 1C), with hepatocyte damage being most pronounced in the HFD + *H. pylori* Cag A + group. Interestingly, hepatocytes in the Cag A- group showed more pronounced vacuolar degeneration than the Cag A + and PBS groups in CD feeding, which may coincide with the significantly heavier liver weights in the Cag A- group than in the other two groups. Histological scoring of MASLD in mice showed that *H. pylori* Cag A + infection aggravated HFD-induced hepatic steatosis more than *H. pylori* Cag A- infection (Fig. 1B).

Glucose homeostasis and insulin sensitivity were tested in the mice by IPGTT and IPITT (Supplementary Fig. 3A, C). HFD-fed mice had significantly weaker glucose regulation capacity than CD-fed mice, and HFD + *H. pylori* Cag A + mice had the weakest glucose regulation. Compared with CD-fed mice, HFD-fed mice showed no significant difference in insulin sensitivity. The serum insulin concentrations and homeostatic model assessment for insulin resistance (HOMA-IR) of the mice in each group are shown in Supplementary Fig. 3B, D.

### *H. pylori* Cag A + infection combined with HFD feeding had the most significant effect on serum metabolism and inflammation in mice

**Fig. 2:**
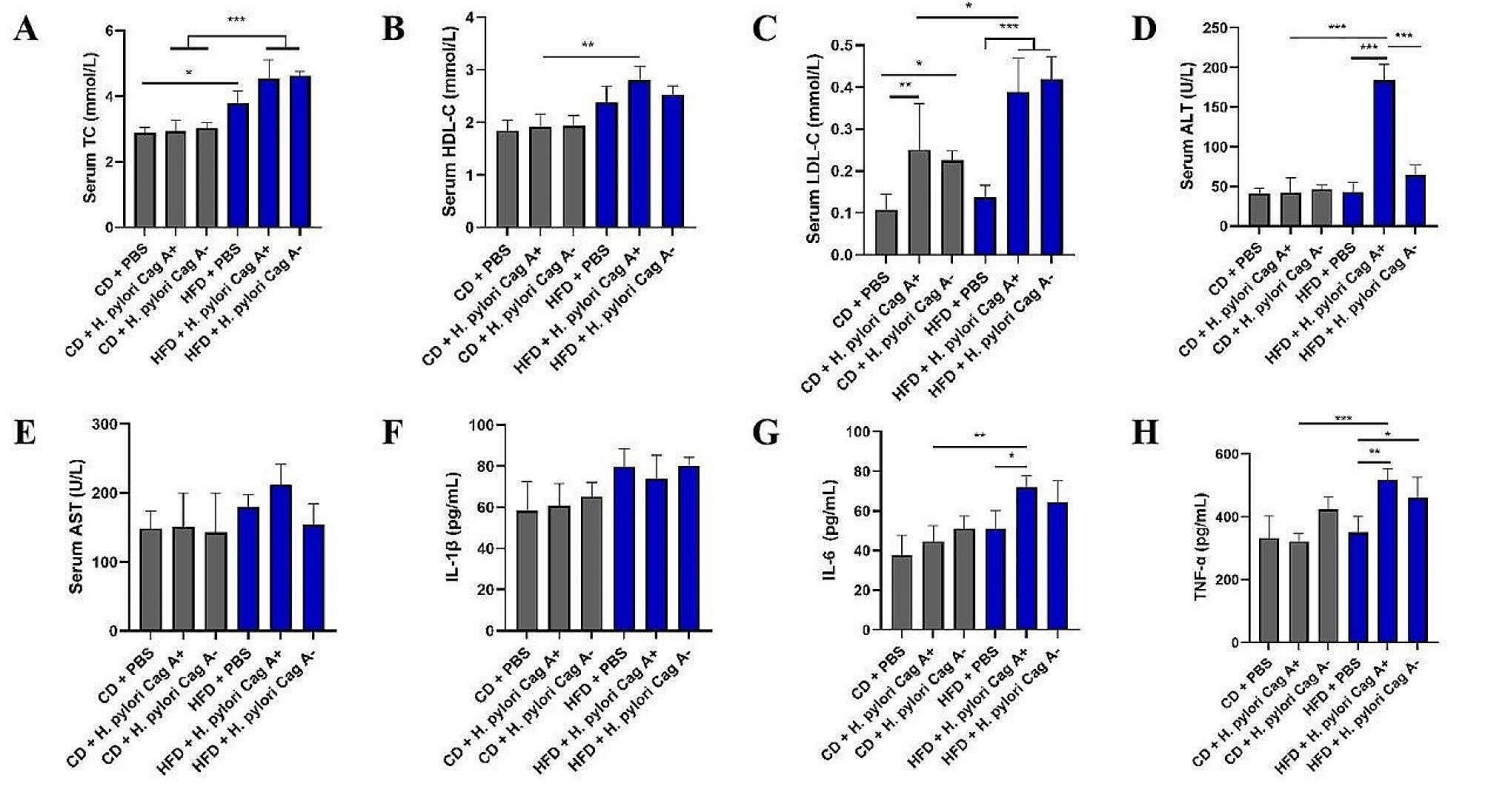
Effects of different *H. pylori* infection combined with CD/HFD feeding on physiological metabolism in mice. (**A**) Serum total cholesterol (TC, mmol/L). (**B**) Serum high-density lipoprotein cholesterol (HDL-C, mmol/L). (**C**) Serum low-density lipoprotein cholesterol (LDL-C, mmol/L). (**D**) Serum alanine aminotransferase (ALT, U/L). (**E**) Serum aspartate aminotransferase (AST, U/L). Serum inflammatory factor: (**F**) IL-1 beta (pg/mL). (**G**) IL-6 (pg/mL). (**H**) TNF-α (pg/mL). Data are expressed as mean ± SD, *n* = 6, *P < 0.05, **P < 0.01, ***P < 0.001

Figure 2 presents the mice’s serum biochemical results and inflammatory factors. Serum TC, ALT, and other results suggested that *H. pylori* Cag A + infection plus HFD feeding significantly affected serum biochemical changes in mice (Fig. 2A-D). The serum inflammatory factors IL-6 and TNF-α had similar results (Fig. 2G, H). In homologous *H. pylori* infection, HFD feeding significantly increased the serum TC and LDL-C levels. However, these changes were not evident in the same diet of different *H. pylori* infected mouse groups. Serum AST and IL-1β levels did not change significantly in any group (Fig. 2E, F).

### RNA-seq reflects different gene expression profiles in the liver of *H. pylori*-infected mice fed with different dietary patterns

**Fig. 3:**
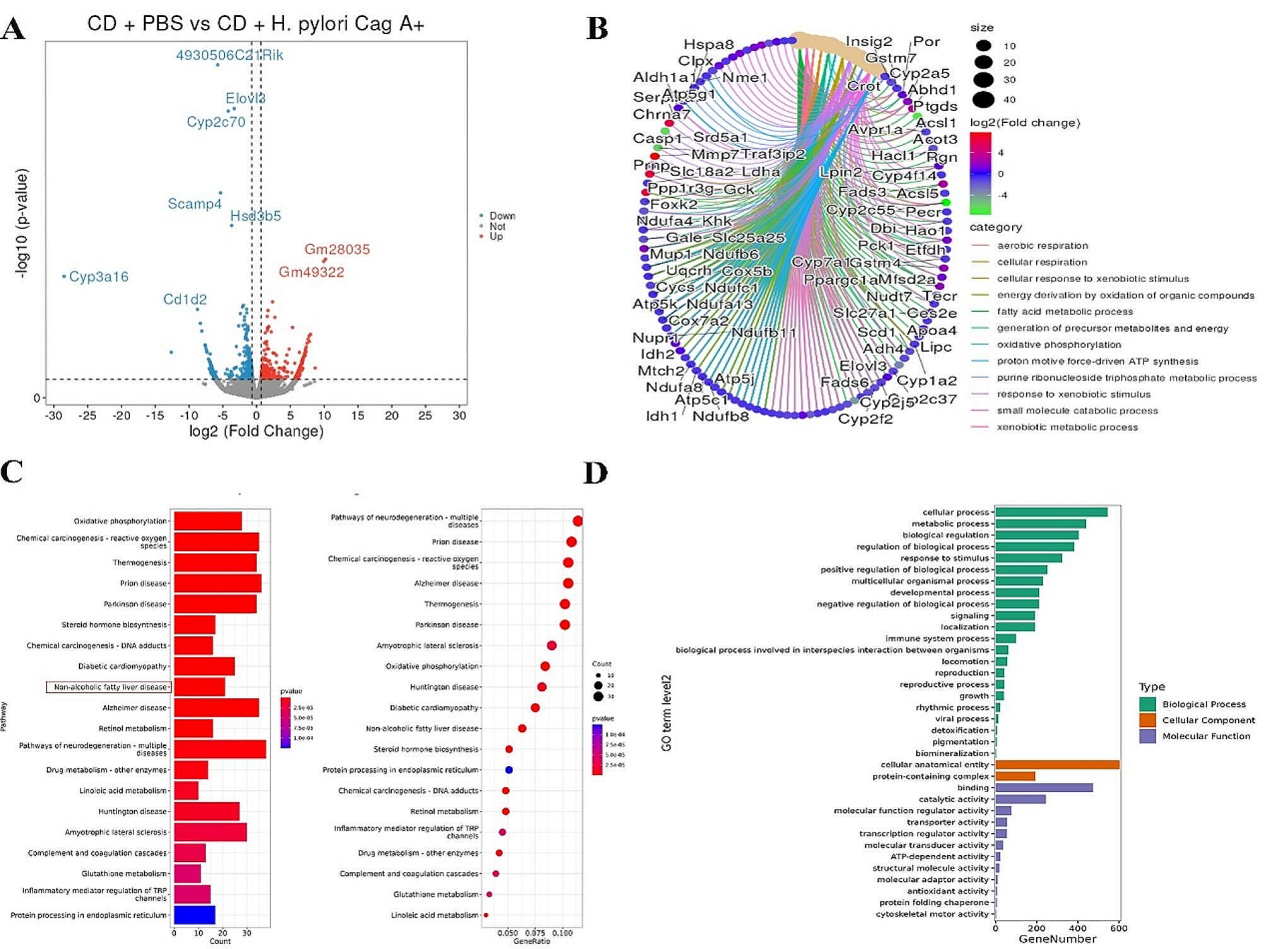
CD + PBS vs. CD + *H. pylori* Cag A + transcriptomic DEGs analysis. (**A**) volcano plot of DEGs; (**B**) DEGs enrichment results visualization, circular cnetplot; (**C**) KEGG enrichment analysis; and (**D**) GO enrichment analysis

Differential expression genes (DEGs) were identified as | fold changes | > 1.5, with a P value < 0.05. Through the analysis of the RNA transcriptome sequencing results of the liver of mice in the CD groups, 767 DEGs were present in the liver of mice in the *H. pylori* Cag A + infected group (Fig. 3A), including 396 down-regulated genes and 371 up-regulated genes. There were 1473 DEGs in the liver of mice infected with *H. pylori* Cag A-, including 750 down-regulated genes and 723 up-regulated genes (Supplementary Fig. 4A). Gene Ontology (GO) and Kyoto Encyclopedia of Genes and Genomes (KEGG) pathway analyses to identify the biological processes associated with the DEGs. Visualization of the DEGs enriched functional results showed that the “fatty acid metabolic process” was significantly expressed (Fig. 3B, Supplementary Fig. 4B). The enrichment results significantly expressed the “Nonalcoholic fatty liver disease” pathway in the KEGG enrichment analysis (Fig. 3C, Supplementary Fig. 4C). In GO enrichment analysis, the top 3 significantly enriched in the biological process were the “cellular process,” “metabolic process,” and “biological regulation (Fig. 3D, Supplementary Fig. 4D).”

**Fig. 4:**
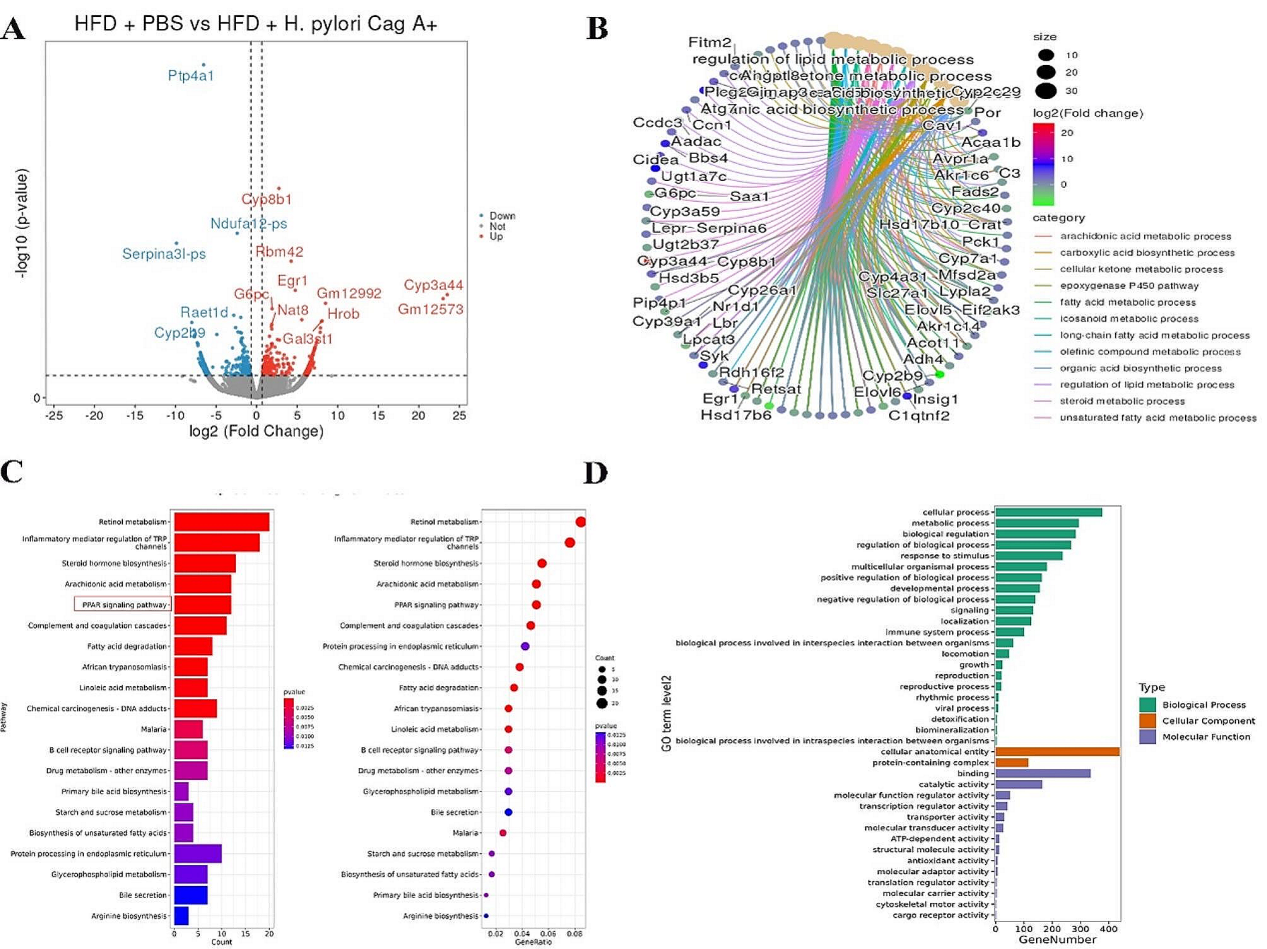
HFD + PBS vs. HFD + *H. pylori* Cag A + transcriptomic DEGs analysis. (**A**) volcano plot of DEGs; (**B**) DEGs enrichment results visualization, circular cnetplot; (**C**) KEGG enrichment analysis; and (**D**) GO enrichment analysis

Analysis of the RNA transcriptome sequencing results of the liver of mice in the HFD groups showed that there were a total of 578 DEGs in the liver of mice infected with *H. pylori* Cag A+ (Fig. 4A), including 245 down-regulated genes and 333 up-regulated genes. Similarly, 452 genes were down-regulated, and 368 genes were up-regulated in the liver of *H. pylori* Cag A- infected mice (Supplementary Fig. 5A). Visualization of the DEGs enriched functional results showed that the “long-chain fatty acid metabolic process” and “regulation of lipid metabolic process " were significantly expressed (Fig. 4B, Supplementary Fig. 5B). In KEGG enrichment analysis, the “PPAR signaling pathway,” “Fatty acid degradation,” and “Retinol metabolism” pathways were significantly expressed in the enrichment results (Fig. 4C, Supplementary Fig. 5C). The GO enrichment analysis results were similar to those of the CD groups, and the top three biological processes were the “cellular process,” “metabolic process,” and “biological regulation (Fig. 4D, Supplementary Fig. 5D).”

### Differential gene expression profiles in livers of *H. pylori*-infected mice with different Cag A status on a uniform diet

**Fig. 5:**
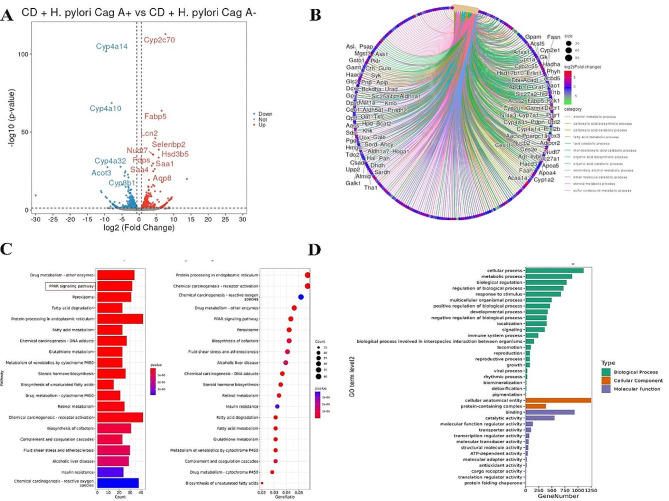
CD + *H. pylori* Cag A + vs. CD + *H. pylori* Cag A- transcriptomic DEGs analysis. (**A**) volcano plot of DEGs; (**B**) DEGs enrichment results visualization, circular cnetplot; (**C**) KEGG enrichment analysis; and (**D**) GO enrichment analysis

In the CD pattern, there were 1511 DEGs in *H. pylori* Cag A- infection compared with *H. pylori* Cag A + infection (Fig. 5A), of which 780 were up-regulated, and 731 were down-regulated. Fatty acid binding protein 5 (Fabp5), a critical intracellular transporter of fatty acid and a key regulator of the PPAR pathway, was significantly up-regulated in the Cag A- group. The visualized DEG enrichment function results showed that the “fatty acid metabolic process” and “fat catabolic process” were significantly expressed (Fig. 5B). The “PPAR signaling pathway” and “Fatty acid degradation” pathways were enriched in KEGG enrichment analysis (Fig. 5C). GO enrichment analysis results are shown in Fig. 5D.

**Fig. 6:**
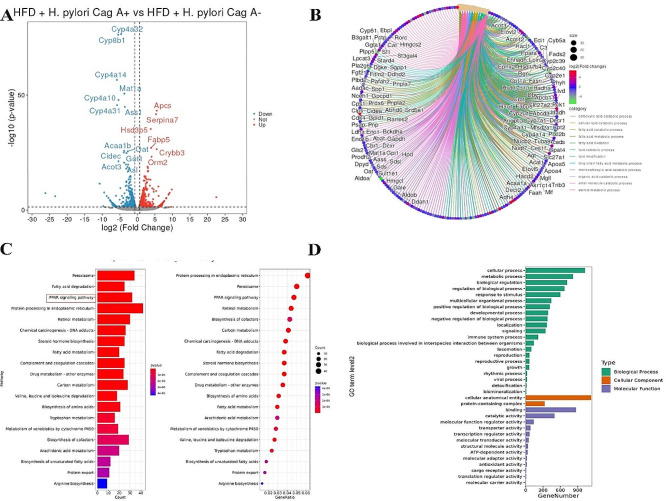
HFD + *H. pylori* Cag A + vs. HFD + *H. pylori* Cag A- transcriptomic DEGs analysis. (**A**) volcano plot of DEGs; (**B**) DEGs enrichment results visualization, circular cnetplot; (**C**) KEGG enrichment analysis; and (**D**) GO enrichment analysis

In HFD feeding, there were 1400 DEGs in *H. pylori* Cag A- infection compared with *H. pylori* Cag A + infection (Fig. 6A), of which 762 were up-regulated, and 638 were down-regulated. Fabp5 was stably and highly expressed in this group. The visualized DEGs enrichment function results showed that “fatty acid oxidation” and “long-chain fatty acids” were significantly expressed (Fig. 6B). The KEGG enrichment analysis enriched the “PPAR signaling pathway” and “Fatty acid degradation” pathways (Fig. 6C). GO enrichment analysis results are shown in Fig. 6D.

### qRT-PCR and IHC verified the expression level of differentially expressed genes in mouse liver

**Fig. 7:**
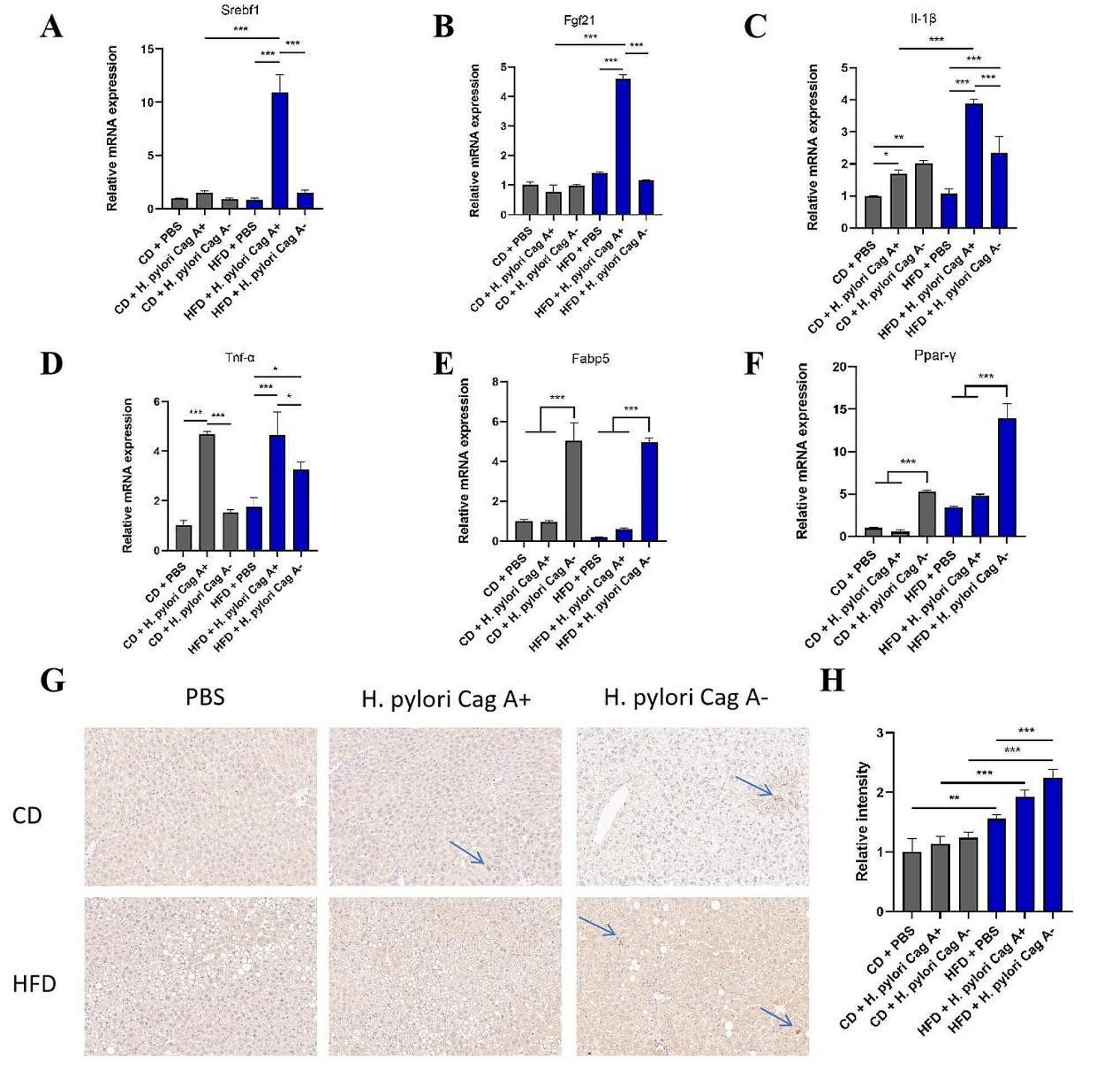
qRT-PCR and IHC validated DEGs expression in mouse liver. Critical lipid metabolism factor Srebf1(**A**), Fgf21 (**B**) inflammatory factor Il-1β (**C**), Tnf-α (**D**). DEGs Fabp5, Ppar-γ were expressed in mouse liver (**E**, **F**). Results of Fabp5 IHC staining in mouse liver in each group (**G**) and quantification bar graph (**H**). 200× magnification under a light microscope. Data are expressed as mean ± SD, *n* = 6, *P < 0.05, **P < 0.01, ***P < 0.001

Subsequently, qRT-PCR results showed that *H. pylori* Cag A + infection combined with HFD significantly increased the expression levels of sterol regulatory element binding transcription factor 1 (Srebf1), fibroblast growth factor 21(Fgf21), the critical factors of lipid metabolism, Il-1β, and Tnf-α in the liver (Fig. 7A-D). We performed qRT-PCR to validate the transcriptome sequencing DEGs, and the validation results are shown in Fig. 7E, F. Meanwhile, For the most significantly differentially expressed Fabp5, we performed IHC staining of the mouse liver. Fabp5 expression levels were higher in *H. pylori* Cag A- infected mouse livers, mainly distributed in interstitial cells. The results were shown in Fig. 7G, H.

## Discussion

Due to the high infection rate of *H. pylori* worldwide and the widespread use of antibiotics, drug resistance is on the rise in some regions. [22]. Additionally, there is an increasing number of patients with MASLD, and a significant proportion of them also have comorbidity with *H. pylori* infection. The direct or potential relationship between the two diseases has been a major focus. A recent meta-analysis demonstrated that *H. pylori* infection was significantly associated with an increased risk of MASLD in both cross-sectional and longitudinal cohort studies [23]. Therefore, there is an expanded benefit for *H. pylori* infection eradication in clinical practice, as it helps clinicians better understand and manage MASLD.

We established mouse models of *H. pylori* infection under different dietary patterns to investigate the association between *H. pylori* infection and MASLD. In the CD groups, *H. pylori* Cag A- infection seemed to cause more hepatocyte damage in mice than Cag A + strain, as measured by liver weight, HE staining, and Oil Red O staining, which was inconsistent with our conjecture. Cag A + strain showed a more significant effect in LDL-C and HOMA-IR. Overall, *H. pylori* infection, regardless of Cag A status, had no noteworthy impact on physiological metabolism, serum biochemical parameters, or liver enzymes in mice. The possible reasons may be: (1) *H. pylori* infection duration is not long enough, the systemic chronic inflammatory response is not apparent, and (2) *H. pylori* infection alone is insufficient to produce significant changes in liver pathology. The analysis of liver transcriptome sequencing results revealed that *H. pylori* Cag A + infection caused 767 DEGs in mouse liver tissues, with 371 up-regulated genes and 396 down-regulated genes. On the other hand, *H. pylori* Cag A- infection led to 1473 DEGs in mouse liver tissues, with 723 genes being up-regulated and 750 genes being down-regulated. Enrichment analysis showed that some DEGs were significantly involved in the “fatty acid metabolism” and “non-alcoholic fatty liver disease” pathway. These transcriptome results illustrate the link between *H. pylori* infection and MASLD.

Based on HFD feeding, *H. pylori* Cag A + infection had a more evident effect on the physiological metabolism of mice. Although there was no statistical difference in body weight and liver weight between the three groups, liver TG content, serum TC, LDL-C, ALT, and serum inflammatory cytokines IL-6 and TNF-α were significantly higher in the *H. pylori* Cag A + infected group than in the other groups. *H. pylori* Cag A + infection combined with HFD feeding resulted in more significant hepatic lipid deposition and hepatocyte macrovesicular steatosis in mice, and there were significant differences in MASLD scores in mice, which coincided with the findings of He et al. [22]. At the same time, mice infected with *H. pylori* Cag A + showed decreased sensitivity to glucose and insulin. Analysis of liver transcriptome sequencing results showed that *H. pylori* Cag A + infection under HFD feeding conditions induced differential expression of 578 genes in mouse liver tissues, of which 245 genes were up-regulated and 333 genes were down-regulated. In the Cag A- *H. pylori* infected group, 452 genes were down-regulated, and 368 were up-regulated in the liver. Enrichment analysis found that some DEGs were significantly involved in the “long-chain fatty acid metabolic process " and “regulation of lipid metabolic process.” Meanwhile, “Retinol metabolism” and “PPAR signaling pathway” were significantly enriched in KEGG analysis. The results of these analyses illustrate that HFD-based *H. pylori* infection impacts hepatic lipid metabolism.

By comparing the effect of *H. pylori* strain infection with different Cag A status on liver transcriptomics under the uniform dietary pattern, we explored the possible role of the virulence factor Cag A in the relationship between *H. pylori* infection and MASLD. The comparison revealed that the “PPAR signaling pathway” and “Fatty acid degradation” pathways were significantly enriched in DEGs from livers of Cag A- *H. pylori*-infected mice regardless of dietary pattern. In addition, Fabp5, a critical regulator of lipid metabolism was upregulated in the transcriptome DEGs.

Several experimental studies have explored the relationship between *H. pylori* and MASLD directly. He et al. reported that *H. pylori* infection combined with 12 weeks of HFD feeding promoted central obesity and IR in mice to a comparable extent as HFD feeding alone for 24 weeks, and dynamic changes in the gut microbiota may cause these effects [24]. Subsequently, the authors measured hepatic lipid deposition in the liver, and MASLD scores revealed that *H. pylori* infection significantly aggravated HFD-induced MASLD and different *H. pylori* strains, most notably SS1, had different exacerbating effects on MASLD [25]. Notably, the *H. pylori* strains used in the above studies (SS1 and NCTC 11,637) did not include the Cag A- strain, and we established a Cag A- strain control in combination with clinical epidemiological studies and *H. pylori* virulence factor studies to explore the effects of different *H. pylori* strains further. In addition, *H. pylori* infection has been demonstrated to promote CCl_4_-induced liver fibrosis in animal models [26]. In this study, it was possible that HFD plus *H. pylori* infection only intervened for 16 weeks, and no significant hepatic fibrosis was observed via Masson staining of liver sections. Combined with the reported literature [27], we estimated that HFD feeding alone requires at least 24 weeks to visualize significant fibrosis in the livers of mice.

Previous studies have confirmed that Cag A is closely related to the occurrence of gastric cancer. Reports on Cag A combined with extra-gastric diseases are common in patients with atherosclerosis [28, 29], and only two studies have reported the association between Cag A and MASLD. Kang et al. suggested that the *H. pylori* Cag A- strain may be associated with MASLD [20]. In contrast, Barreyro et al. reported no significant association between *H. pylori* infection, Cag A status, and ultrasonographically diagnosed MASLD in MASLD patients with dyspeptic symptoms [30]. Moreover, the results suggested that Cag A + but not Cag A- was associated with higher AST and fibrosis 4 scores in patients. Our study is the first transcriptomical research to mechanistically explore the relationship between Cag A, *H. pylori*, and MASLD. Sequencing analysis of liver transcriptomes infected with different *H. pylori* strains revealed that “Nonalcoholic fatty liver disease” and “PPAR signaling pathway” were enriched according to KEGG enrichment analysis, and Fabp5 expression was significantly higher in the Cag A- groups.

Fabp5 is a member of the fatty acid binding protein family, which is mainly involved in the uptake, transport, and metabolism of fatty acids and related metabolites in the cytoplasm and regulating lipid metabolism and cell growth [31]. Fabp5 is essential for the pathogenesis of IR associated with obesity and lipid metabolism [32, 33]. Loss of Fabp5 gene expression leads to increased systemic insulin sensitivity in animal models of obesity and IR, and adipocytes isolated from Fabp5 -/- mice also exhibit increased insulin-stimulated glucose transport capacity [32]. In contrast, mice with high Fabp5 expression in adipose tissue exhibited significantly decreased systemic insulin sensitivity, and Fabp5 may regulate blood glucose and blood lipid metabolism by affecting leptin expression.

In this study, we detected the differential expression of Fabp5 in each group by qRT-PCR. Interestingly, Fabp5 was highly expressed in both the Cag A- groups but not in the Cag A + groups, regardless of the dietary ingredient. In addition, two bioinformatics studies predicted the crucial role of Fabp5 in MASLD. High Fabp5 expression was significantly associated with poor prognosis in MASLD-related HCC patients [34, 35]. These results fit our results to some extent. Therefore, we speculated that other virulence factors of *H. pylori*, such as vacuolating toxin A, neutrophils activating protein, upregulated Fabp5 expression through some mechanisms, while the presence of Cag A, the most potent virulence factor, masked the mechanisms. However, additional experimental studies are needed to explore the underlying mechanisms of *H. pylori* virulence factors and extragastric diseases such as MASLD.

The enrichment analysis results suggested that the retinol metabolic and PPAR signaling pathways were significantly enriched in the HFD groups. Retinol and its primary metabolites, retinal and all-trans retinoic acid (atRA), are collectively referred to as naturally occurring retinoids, which control energy balance, obesity, and inflammatory processes. Total cellular reflectance retinoic acid binding protein (CRABP) is the primary receptor for intracellular retinoid transport, and Fabp5 also has a high affinity for atRA and long-chain fatty acids. Fabp5 competitively binds atRA with CRABP2, and when the Fabp5/CRABP2 ratio is high, atRA binds Fabp5 and activates the downstream PPAR pathway, a crucial pathway regulating glucose and lipid metabolism [36, 37]. We speculate that the overexpression of Fabp5 in mouse hepatocytes caused by *H. pylori* infection inhibits CRABP2, binds to atRA for transport, activates the downstream PPAR pathway, and, in turn, regulates fatty acid degradation pathways. Our experimental results concatenate *H. pylori* infection exacerbating MASLD into a complete clue.

Compared with the published research in the field [24, 25], our study directly explored the effects of *H. pylori* virulence factor Cag A on the physiological metabolism and liver pathology of mice and reported the transcriptomic changes in the liver of mice infected with *H. pylori* for the first time, which contributed to the further study of the mechanism of *H. pylori*-induced extra-gastric diseases and the exploration of the direct link between *H. pylori* and MASLD. However, this study also has some limitations. First, in this study, the HFD feeding time was 16 weeks, and Masson staining showed no significant fibrosis of the liver matrix. If we want to investigate *H. pylori* infection and MASH-related liver fibrosis and HCC, we need to extend the intervention time further. Second, the two groups of *H. pylori* used in this study took the most significant virulence factor, Cag A status difference, when compared, ignoring other minor differences in the genome between the two groups. In the future, prolonging experimental intervention time can better simulate the status of chronic *H. pylori* infection with MASLD in humans, and the construction of specific virulence factor knockout of *H. pylori* can better study the specific mechanism of *H. pylori* and MASLD.

## Conclusion

In summary, we established a mouse model of MASLD plus *H. pylori* infection and found that chronic *H. pylori* infection significantly aggravated HFD-induced hepatic lipid deposition and IR. Through transcriptome sequencing analysis and related validation, we discovered that *H. pylori* infection may promote the development of MASLD by regulating lipid metabolism. However, how does *H. pylori* infection regulate Fabp5 expression in the liver, through hepatic macrophages? *H. pylori* exoteric vesicles? Or other pathways. Basic experiments are needed to explore the underlying mechanisms involved in MASLD, which will help us better comprehend MASLD and gain insight into the pathways through which *H. pylori* causes extra-gastric diseases.

### Electronic supplementary material

Below is the link to the electronic supplementary material.

Supplementary Material 1

## Data Availability

The datasets generated and/or analyzed during the current study are available from the corresponding author upon reasonable request.

## Abbreviations

*H. pylori*: *Helicobacter pylori*
MASLD: Metabolic dysfunction-associated steatotic liver disease
Cag A: Cytotoxin-associated gene A
Fabp5: Fatty acid binding protein 5
PPAR: Peroxisome proliferator-activated receptor
IR: Insulin resistance
HOMA: IR-Homeostatic model assessment for insulin resistance
FBG: Fasting blood glucose
TG: Triglyceride
BMI: Body mass index
HDL-C: High-density lipoprotein cholesterol
LDL-C: Low-density lipoprotein cholesterol
HFD: High-fat diet
CD: Chow diet
ALT: Alanine aminotransferase
AST: Aspartate aminotransferase
TC: Total cholesterol
IPGTT: Intraperitoneal glucose tolerance test
IPITT: Intraperitoneal insulin tolerance test
KEGG: Kyoto Encyclopedia of Genes and Genomes
GO: Gene Ontology
DEG: Differentially expressed gene
IL-1β: Interleukin 1β
TNF-α: Tumor necrosis factorα
IL-6: Interleukin 6
atRA: All-trans retinoic acid
