# Supplementary material for: Helicobacter pylori infection exacerbates metabolic dysfunction-associated steatotic liver disease through lipid metabolic pathways: a transcriptomic study
Source: J Transl Med. 2024 Jul 29;22:701. doi: 10.1186/s12967-024-05506-y (PMC11288106; doi:10.1186/s12967-024-05506-y)
Supplement: Supplementary file 1 — Supplementary Material 1 [file 12967_2024_5506_MOESM1_ESM.docx]

**Supplementary Figure:**

**
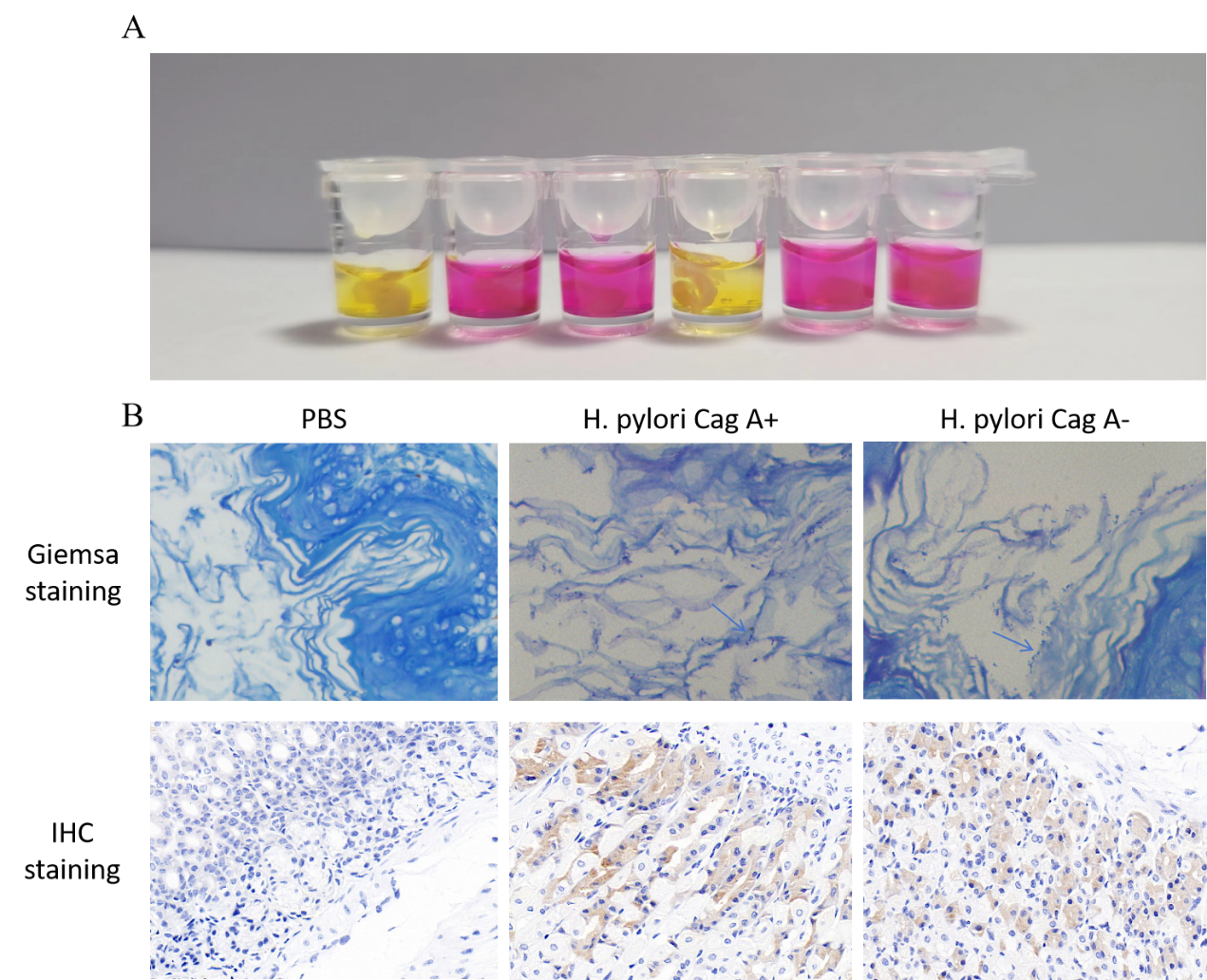
**

Supplementary Figure 1 Validation results for *H. pylori* infection in mice. (A) Rapid urease test of mouse gastric mucosa, sequential numbers of samples from left to right were CD + PBS; CD + H. pylori Cag A +; CD + H. pylori Cag A −; HFD + PBS; HFD + H. pylori Cag A +; HFD + H. pylori Cag A −. A positive result is a plum red or purple color of the assay. (B) Giemsa staining and IHC of *H. pylori* antibody in mice gastric mucosa, 400x under a light microscope.

**
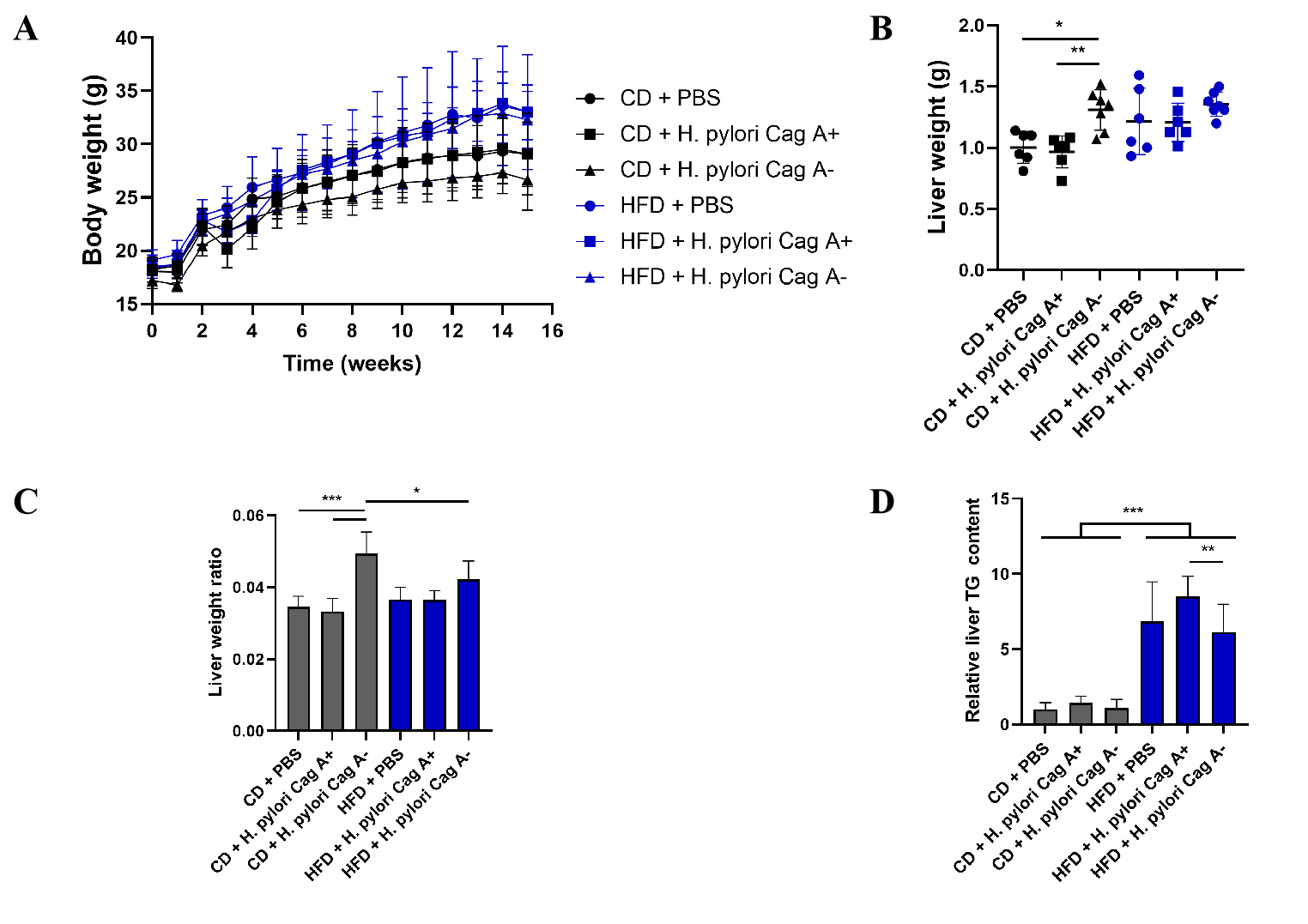
**

Supplementary Figure 2 Effects of *H. pylori* infection combined with CD/HFD feeding on body and weight in mice. Body weight curves (A), Liver weight (B), Liver weight ratio: liver weight/body weight × 100% (C), Relative TG content in mice liver in CD/HFD groups (D).


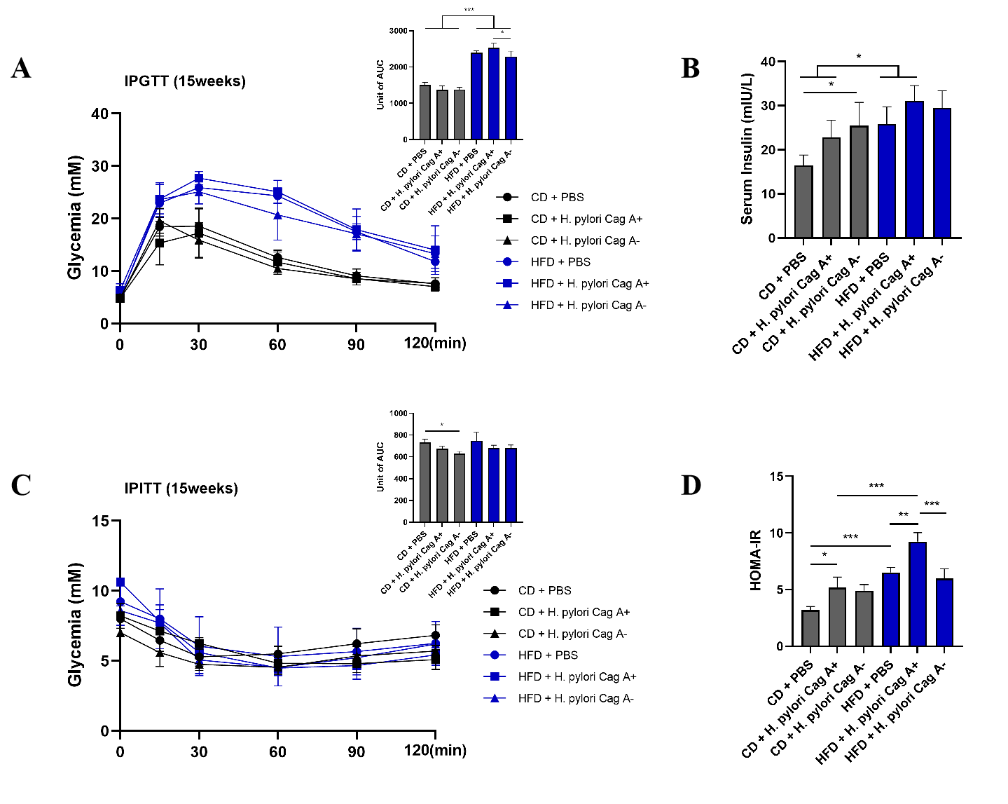


Supplementary Figure 3 Effect of *H. pylori* infection combined with CD/HFD feeding on mouse glucose homeostasis and insulin sensitivity. In the 15th week of intervention, the mice were subjected to the IPGTT (A) and IPITT (C), and the AUC measurements are shown in the right upper corner of the picture. Serum insulin levels (mmol/L) in CD/HFD groups (B). Insulin resistance was calculated: HOMA-IR = fasting plasma glucose (mmol/L) × fasting insulin (μU/mL)/22.5. HOMA-IR in CD/HFD groups (D). Data are expressed as mean ± SD, n=6, *P<0.05, **P<0.01, ***P<0.001.


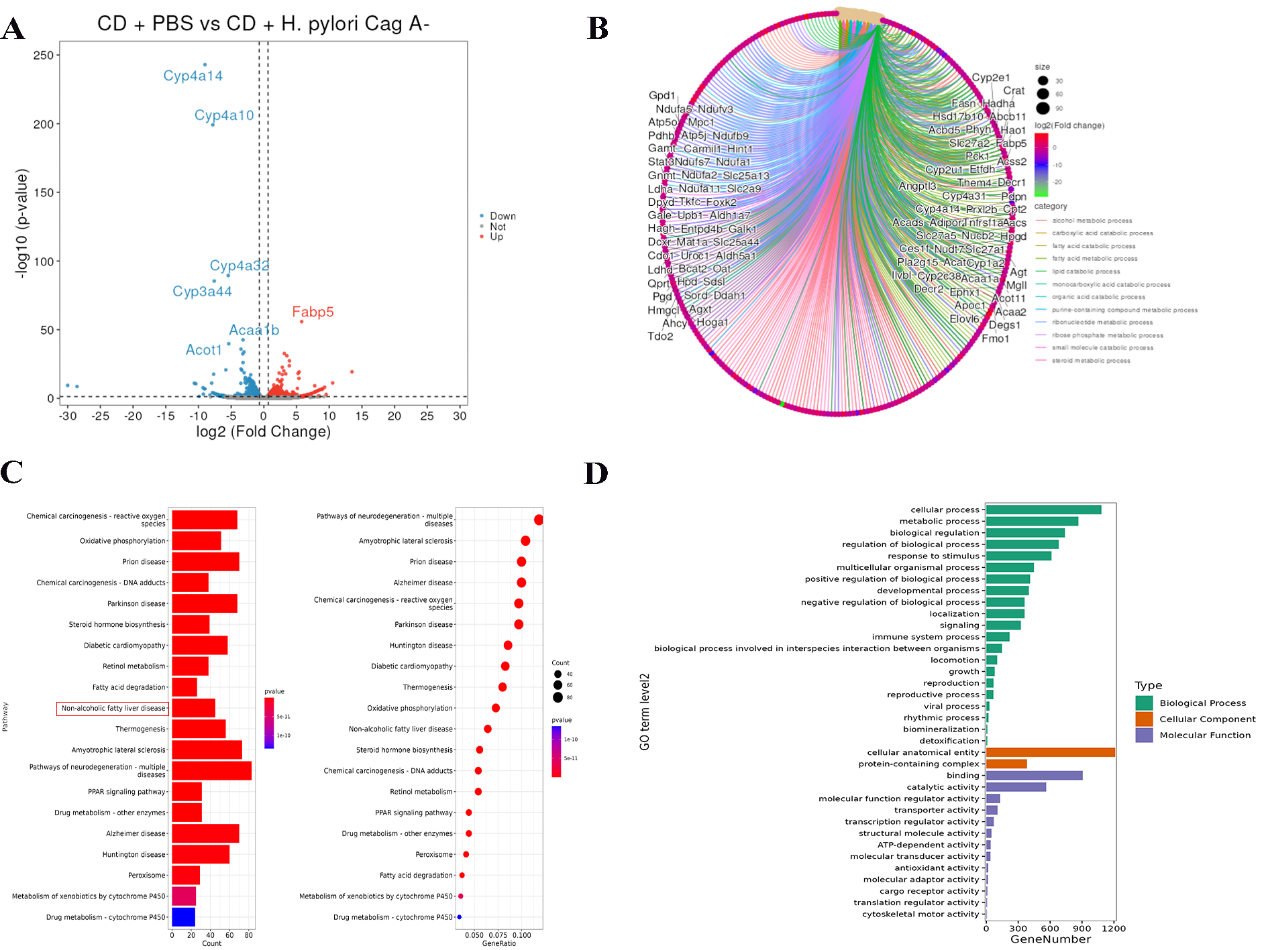


Supplementary Figure 4 CD + PBS vs CD + H. pylori Cag A- transcriptomic DEGs analysis. (A) volcano plot of DEGs; (B) DEGs enrichment results visualization, circular cnetplot; (C) KEGG enrichment analysis; and (D) GO enrichment analysis.


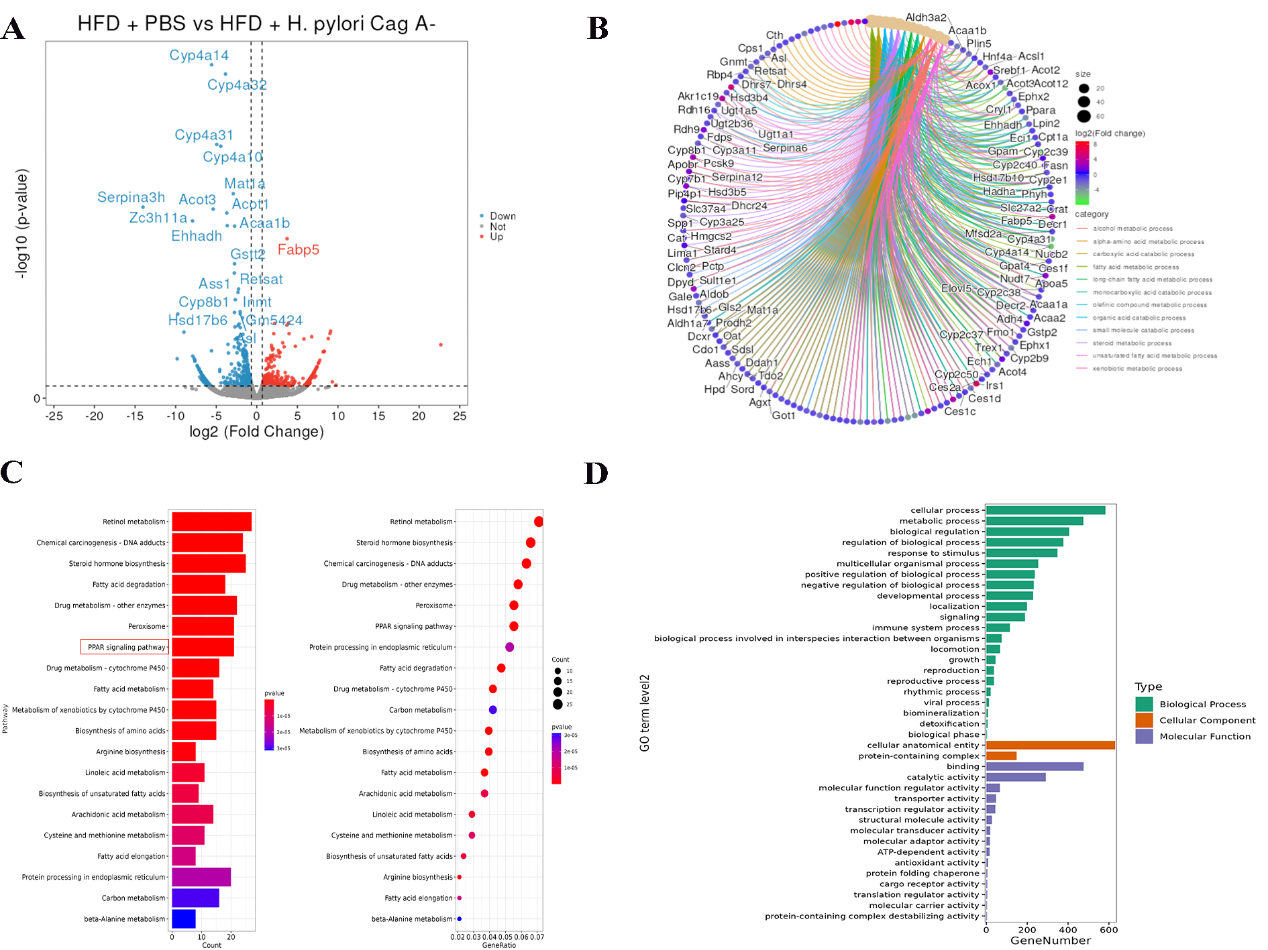


Supplementary Figure 5 HFD + PBS vs HFD + H. pylori Cag A+ transcriptomic DEGs analysis. (A) volcano plot of DEGs; (B) DEGs enrichment results visualization, circular cnetplot; (C) KEGG enrichment analysis; and (D) GO enrichment analysis.
